# The ING tumor suppressors in cellular senescence and chromatin

**DOI:** 10.1186/2045-3701-1-25

**Published:** 2011-07-18

**Authors:** Susann Ludwig, Alexandra Klitzsch, Aria Baniahmad

**Affiliations:** 1 Institute of Human Genetics, Jena University Hospital, D-07743 Jena, Germany

## Abstract

The Inhibitor of Growth (ING) proteins represent a type II tumor suppressor family comprising five conserved genes, *ING1 *to *ING5*. While ING1, ING2 and ING3 proteins are stable components of the mSIN3a-HDAC complexes, the association of ING1, ING4 and ING5 with HAT protein complexes was also reported. Among these the ING1 and ING2 have been analyzed more deeply. Similar to other tumor suppressor factors the ING proteins are also involved in many cellular pathways linked to cancer and cell proliferation such as cell cycle regulation, cellular senescence, DNA repair, apoptosis, inhibition of angiogenesis and modulation of chromatin.

A common structural feature of ING factors is the conserved plant homeodomain (PHD), which can bind directly to the histone mark trimethylated lysine of histone H3 (H3K4me3). PHD mutants lose the ability to undergo cellular senescence linking chromatin mark recognition with cellular senescence. ING1 and ING2 are localized in the cell nucleus and associated with chromatin modifying enzymes, linking tumor suppression directly to chromatin regulation. In line with this, the expression of ING1 in tumors is aberrant or identified point mutations are mostly localized in the PHD finger and affect histone binding. Interestingly, ING1 protein levels increase in replicative senescent cells, latter representing an efficient pathway to inhibit cancer proliferation. In association with this, suppression of p33ING1 expression prolongs replicative life span and is also sufficient to bypass oncogene-induced senescence. Recent analyses of ING1- and ING2-deficient mice confirm a tumor suppressive role of ING1 and ING2 and also indicate an essential role of ING2 in meiosis.

Here we summarize the activity of ING1 and ING2 as tumor suppressors, chromatin factors and in development.

## Association of ING factors with cellular senescence

The ING proteins are evolutionary conserved factors with orthologs identified from yeast to human [1]. In mouse and human the five different *ING *loci are distributed over the genome, most of which give rise to multiple protein products as a consequence of alternative splicing events [2]. The *ING1 *gene harbors three exons and can be alternatively spliced to generate p47ING1a, p33ING1b, p27ING1d, and p24ING1c in human [2]. Among these isoforms p33ING1b is the most widely expressed in human tissues. Mouse *ING1 *was found to have three splice variants, p31ING1a, p31ING1c and p37ING1b [3]. So far identified, *ING2 *encodes two isoforms, ING2a (p33ING2) and ING2b [4].

Decreased ING1 expression or loss of heterozygosity of the *ING1 *locus have been observed in various tumors, including breast, ovarian, head carcinomas and melanoma [5,6]. Also, ING2 displays reduced expression levels in different tumors, including lung cancer and melanoma [6].

The role of ING1 and ING2 as tumor suppressor proteins is supported since both factors are involved in cellular senescence, an effective anti-tumor pathway. Senescent cells are characterized by a stable and irreversible cell cycle arrest, frequently in the G_0_/G_1 _phase of the cell cycle [7]. Although they do not divide, senescent cells are viable, metabolically active and do not engage in programmed cell death (apoptosis) [7]. The senescent phenotype can be induced by a variety of stimuli. Replicative senescence is presumably triggered by telomere shortening, whereas premature senescence can be activated in response to a variety of stress signals, including overexpression of oncogenes such as *Ras *or e.g. oxidative DNA damage [7,8].

Many signaling pathways are responsible for the implementation and maintenance of the senescent phenotype caused by different stimuli, including changes in expression levels of Cyclin D1, c-Myc-Bmi, promyelocytic leukemia protein (PML), p53 or retinoblastoma protein (pRb) [2]. Cellular senescence is accompanied by specific epigenetic changes which play an important role in regulation of gene expression and appearance of heterochromatic domains known as senescence-associated heterochromatic foci (SAHFs) [9,10].

First evidence for ING1 to be involved in cellular senescence was shown by RNA and protein levels of p33ING1 that were 8- to 10-fold higher in senescent cells compared to young, proliferation-competent human diploid fibroblasts [11]. Accordingly, expression of antisense *ING1 *RNA resulted in an extension of replicative life span of normal human fibroblasts, suggesting an important role for p33ING1 in the initiation of replicative senescence [11]. In addition, ectopic expression of p33ING1 in primary human fibroblasts resulted in growth arrest with an expression of senescence-specific markers, e.g. senescence-associated β-galactosidase activity [12]. Furthermore, it was shown that p33ING1 mRNA-level was up-regulated in senescent human prostate epithelial cells [13]. In line with this, inactivation of p33ING1 promoted neoplastic transformation [14]. Other reports concerning the isoform p47INGa have shown that the protein level of p47INGa increased during replicative senescence of human fibroblasts [15]. In addition, transient overexpression of the p47ING1a isoform was able to induce a senescent phenotype in the same cell type [15]. Taken together, these observations suggest that both isoforms can play a role in the implementation of replicative senescence.

Studies concerning oncogene-induced senescence revealed that suppression of p33ING1 by RNA-interference in human fibroblasts was sufficient to bypass oncogene-induced senescence [16]. In line with this, p33ING1 protein level was increased in senescent human fibroblasts, whereas p47ING1a protein levels did not change [17]. These observations suggest an important role for p33ING1 in the implementation of oncogene-induced senescence.

Analyses of mouse embryonic fibroblasts (MEFs) which exhibited a reduced expression of all murine *ING1 *transcripts showed impaired induction of senescence triggered by oncogenic stress [18]. This also supports a role for the Ing1 locus as a critical mediator of the oncogene-induced senescence. In contrast, p37ING1-deficient but p31ING1-expressing MEFs did not exhibit an altered oncogene-induced senescence in comparison to wildtype MEFs [19]. This suggests that the ING1 isoforms might have specific roles in the implementation of oncogene-induced senescence in MEFs.

There is also a functional link of ING proteins to p53 in senescence as regulators of p53 protein stability and posttranslational modification [2,20]. An increase of p53 transcriptional activity can also be associated with cellular senescence [21]. Recently, it has been shown that human fibroblasts with a disabled p53 pathway and enforced p33ING1b expression failed to trigger efficiently cellular senescence [16], suggesting that the implementation of cellular senescence by p33ING1 requires an intact p53 pathway.

However, studies analyzing ING1-deficient MEFs indicated p53-independent pathways of ING1-mediated cellular senescence [19,22]. Taken together, these results suggest that the link between ING1 and p53 in the implementation of cellular senescence might be cell type specific.

**Figure 1:**
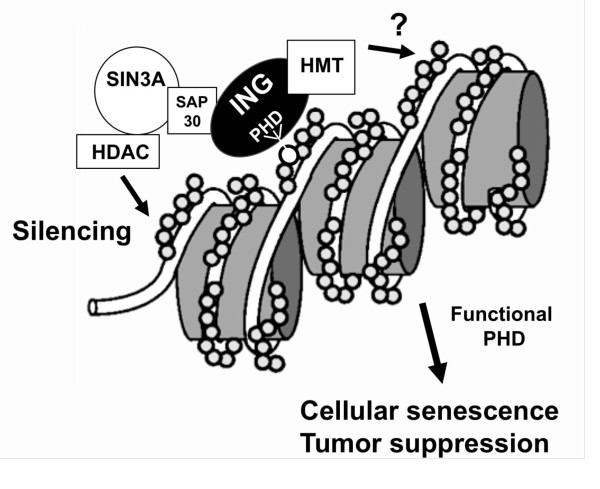
**The functional role of the ING- Plant Homeodomain (PHD)**. The role of the PHD was analyzed by mutations of this domain. The PHD is essential for binding to histone H3 at trimethylated lysine 4, the induction of cellular senescence and tumor suppression.

Interestingly, human fibroblasts transfected with p33ING1 carrying point mutations in the PHD, which led to deficient H3K4me3 binding, were not able to undergo cellular senescence [16]. This strengthens the role of ING1 as tumor suppressor and suggests an important role of the PHD and chromatin in regulation of cellular senescence (Figure 1). Notably, p33ING1 was found to accumulate on chromatin in oncogene-induced senescent human fibroblasts, but is excluded from SAHFs as it was also for the histone mark H3K4me3 [16]. This supports a critical role of ING1 protein in the induction of cellular senescence, whereas it remains to be shown whether ING1 is required for maintenance of the irreversible cellular senescence status.

The role of ING2 in the regulation of cellular senescence indicates a more complex situation. Overexpression of ING2 in early passage human fibroblasts led to an induction of premature senescence in a p53-dependent fashion, whereas knockdown of ING2 with a small interfering RNA construct delayed onset of replicative senescence in these cells. In high passaged cells, an increased p33ING2 expression has been reported [23]. However, other *in vitro *studies analyzing cellular senescence suggest a functional interplay of ING2 with the p53 tumor suppressor in two different manners. Down-regulation of ING2 in human telomerase reverse transcriptase (hTERT)-immortalized human fibroblasts by small interfering RNA was associated with a senescence-like arrest, which occurred in a p53-independent manner. In contrast, overexpression of ING2 was found to induce a p53-dependent senescence pathway [24]. The different observations might be linked to the different cell types and noteworthy may link hTERT expression to ING2 activity.

Interestingly, analyses of the ING2-isoforms, demonstrated different expression levels of ING2a and ING2b in human fibroblasts undergoing replicative senescence. It was found that the expression level of the ING2-isoform, ING2a, which is closely related to p33ING1, was down-regulated in a p53- dependent manner in these cells during replicative senescence. In contrast, expression level of the shorter isoform ING2b was not changed in senescent cells in comparison to the expression level in young human fibroblasts [25]. This indicates that the expression of ING2a and ING2b might be differentially regulated and be important in determining cell fate.

## Association of ING1 and ING2 with chromatin

p33ING1 and ING2 are purified as stable components of the SAP30 - mSIN3a histone deacetylase (HDAC) complexes and are also shown to be associated with brahma-related gene 1 (Brg1)-based SWItch/Sucrose NonFermentable (SWI/SNF) chromatin remodelling complexes [26,27].

The abilities to bind to histone marks and to modify histones are significant features of ING proteins. The conserved PHD of ING1 and ING2 binds to histone 3 di- or trimethylated at lysine 4 (H3K4me2, H3K4me3) which is a histone mark associated with activated and open chromatin [28]. The binding of the PHD to H3K4me2/3 was shown to occur after genotoxic stress and in response to DNA damage. Mutations within the PHD prevent this interaction and are associated with reduced DNA repair after UV-irradiation or promote DNA-damage induced apoptosis in melanoma cells [29,30]. The PHD of ING1 and ING2 is also associated with a histone methyl transferase activity (HMT) that methylates histones H1 and H3 residues, within histone H3 residues the first 20 amino acids, however excluding the common methyl acceptor sites lysine 4 and lysine 9 [31].

Furthermore, the N-termini of ING1 and ING2 directly interact with Sin3A-associated protein (SAP30), a component of the Sin3 homolog A/HDAC (Sin3A/HDAC) repressor complex [32] linking HMT and HDAC activity to the activating histone mark H3K9me3. This also suggests that the interaction of the PHD to H3K4me2/3 stabilizes the Sin3A/HDAC repressor complex at the nucleosomes for the deacetylation of histone residues linking DNA repair, apoptosis and tumorigenic functions with chromatin regulation and gene transcription [32].

Interestingly, binding assays in HeLa cells have shown that p33ING1 and ING2 also recruit Sirtuin1 (SIRT1) to the Sin3A/HDAC complex [33]. SIRT1 uses Nicotinamide adenine dinucleotide (NAD+) as a cofactor providing a possible link between metabolic pathways and epigenetic control [34].

The interaction between ING1 and the Sin3A/HDAC complex plays also an important role in the maintenance of genome stability in mammalian cells [35,36]. Studies by Xin *et al*. (2004) in HeLa cells showed that p33ING1 interacts with Sin3A/HDAC complex and the DNA methyltransferase 1 -associated protein 1 (DMAP1) to maintain hypoacetylation and histone methylation in pericentric heterochromatin during late S phase of the cell cycle [35,36]. Taken together, these results indicate that ING proteins are involved in cellular processes connecting histone deacetylation, histone methylation, and DNA methylation in maintaining pericentric heterochromatin structure throughout cell divisions [37].

Another link to gene repression was revealed by findings that ING1 and ING2 interact with the corepressor Alien *in vitro *and *in vivo *[2,38]. Alien is a corepressor for certain nuclear hormone receptors and was also found as a corepressor for cell cycle regulator E2F1, inhibiting its transcriptional activity [39,40]. Interestingly, Alien binds Sin3A as well and mediates gene silencing in part by the recruitment of HDAC activity [39]. The interaction with ING1 and ING2 was shown to enhance the Alien-mediated gene repression [38]. Thus, ING1 and ING2 bind to methylated histone H3 and recruit HDAC and HMT activity associated with gene repression.

Interestingly, other studies showed an ING1 isoform specific interaction with histone acetyl transferases (HATs). The interaction of p33ING1 with HATs was shown to lead to a hyperacetylation of histones H3 and H4, whereas the p47ING1 isoform inhibits acetylation of these histones H3 and H4 [2,41,42]. In addition, p33ING1 seems to play a tethering role between p300 and proliferating cell nuclear antigen (PCNA) facilitating their physical functions, p300-mediated chromatin remodelling and PCNA function in DNA repair. Studies revealed that p33ING1b is complexed with both p300 and PCNA [41]. The ING1-PCNA interaction was observed following DNA damage after UV-irradiation. In contrast, ING1 mutants lacking the PCNA interaction failed to induce apoptosis after UV-irradiation. This may suggest that the ING1-PCNA-HAT interaction may occur in cells in stress-mediated apoptosis induction [41].

Moreover, ING2 also interacts with HATs leading to a p300-mediated p53 acetylation, which results in an active p53 state, although ING2 cannot directly bind to p53 [23,43]. This suggests that ING2 initiates an enhanced binding of p53 to p300, acting as a cofactor for p300-mediated p53 acetylation [23,43]. The ING1 and p53 interaction may be involved in DNA repair and genome stability. After UV-irradiation ING2 mediates the recruitment of the damage-recognition *Xeroderma pigmentosum *group A-complementing (XPA) protein to the photo-lesion site. In line with this, ING2-knockdown suggests ING2 as an essential factor for the nucleotide excision repair. Thus, ING2 seems to negatively regulate cell proliferation through activation of p53 by enhancing its acetylation and is required for the initial DNA damage sensing and chromatin regulation in the nucleotide excision repair process [44].

## Developmental role of ING1 and ING2

The developmental and physiological functions of ING1 and ING2 *in vivo *have been investigated by manipulating endogenous ING genes. Several mouse models with deficiency in the *ING1 *locus have been described [19,22]. Mouse *ING1 *was found to have three splice variants, p31ING1a, p31ING1c and p37ING1b [3]. Kichina *et al*. (2006) used gene targeting embryonic stem cells to generate Ing1-null mice. These mice were viable and approximately 20% smaller in size compared to their wild-type littermates. Notably, the Ing1-null mice were more prone to effects of whole body irradiation. Interestingly, while none of the age-matched wild-type littermates developed B-cell lymphoma, a significant number of aged ING1 knock-out mice developed this type of tumor. Consistent with this observation, mice deficient for specifically the p37ING1 predominant isoform also developed spontaneous follicular B-cell lymphoma [19]. This indicates that the p37ING1 isoform may play a role in the suppression of B-cell lymphoma *in vivo*, which is in line with the role of ING1 as a tumor suppressor.

Moreover, the role of ING1 expression patterns in mammalian development was analyzed in 12.5-day mouse embryos. These observations suggest a possible involvement of ING1 in developmental regulated apoptosis because high levels of ING1 were observed in regions, which are known for increased apoptosis during embryogenesis [45].

It is noteworthy that studies of Coles *et al*. (2007) and Kichina *et al*. (2006) suggest no correlation between p53 and ING1 proteins, indicating a p53-independent role for ING1 in the regulation of cell growth and apoptosis *in vivo*.

Comparing ING1- and ING2-deficient mice indicates that not all ING-mediated functions are redundant. The developmental and physiological functions of ING2 through targeted germline disruption suggest that ING2-deficient mice exhibited a normal body size [46], whereas ING1-deficient mice were smaller compared to the wild-type mice [22]. Interestingly, loss of ING2 resulted in an elevated incidence of soft-tissue sarcomas, while ING1-deficient mice preferentially developed B-cell lymphomas, supporting the role of ING proteins as tumor suppressors [22,46]. ING2 expression was abundant in testes of normal mice and the testes of ING2-deficient mice showed a degeneration of seminiferous tubules and meiotic arrest before pachytene stage with incomplete meiotic recombination. Consequently, this defect in spermatogenesis of ING2-deficient mice led to male infertility indicating an essential role for ING2 in meiosis [46]. On the other hand, ING1 expression was low in testes [47] and ING1-deficient mice seemed not to exhibit any abnormalities in spermatogenesis and were fertile [22]. Taken together, these studies provide evidences that endogenous ING1 and ING2 seem to have diverse functions in tumor suppression and spermatogenesis.

## Conclusions

ING1 and ING2 are interesting factors due to their role in a battery of cellular protective pathways. This seems to be mediated by interaction with other cellular factors. An important role seems to be mediated by the PHD, which links histone H3K4me3 to ING-regulated pathways. However, the detailed roles of ING1 and ING2 are not fully solved yet. It is unclear whether ING factors are required only for induction of cellular senescence or also for its maintenance. It is also unclear under which control ING interacts in a pathway-specific manner with other factors. Also, the posttranslational modifications of ING that have regulatory roles require to be addressed. The potential redundant roles of ING1 and ING2 on developmental level will provide further insights by generating double null mutants.

## Abbreviations

Brg1: brahma-related gene 1; DMAP1: DNA methyltransferase 1-associated protein 1; HAT: histone acetyl transferase; HDAC: histone deacetylase; HMT: histone methyltransferase; hTERT: human telomerase reverse transcriptase; H3K4me3: trimethylated lysine of histone H3; ING: inhibitor of growth; MEFs: mouse embryonic fibroblasts; NAD+: Nicotinamide adenine dinucleotide; PCNA: proliferating cell nuclear antigen; PHD: plant homeodomain; PML: promyelocytic leukemia protein; pRb: retinoblastoma protein; SAHFs: Senescence-Associated Heterochromatic Foci; SAP30: Sin3A-associated protein, 30 kDa; Sin3A: Sin3 homolog A; SIRT1: Sirtuin1; SWI/SNF: SWItch/Sucrose NonFermentable; XPA: Xeroderma pigmentosum group A-complementing protein.

## Competing interests

The authors declare that they have no competing interests.

## Authors' contributions

All authors read and approved the final manuscript.
